# Fabrication and mechanical properties of porous tantalum carbon composites by chemical vapor deposition

**DOI:** 10.1038/s41598-025-86680-x

**Published:** 2025-01-21

**Authors:** Junyu Zhu, Wenting Li, Hongzhong Cai, Xian Wang, Xingqiang Wang, Wuxun Zhu, Yan Wei, Xuming Li, Xingdong Zhao, Guixue Zhang, Haohong Jiang

**Affiliations:** 1 Kunming Institute of Precious Metals, Kunming, 650106 China; 2 School of Civil Engineering, Central South University, Changsha, 410083 China

**Keywords:** CVD, Carbon foam, Ta coatings, Uniformity, Mechanical properties, Materials science, Materials for energy and catalysis

## Abstract

This study investigates the deposition of tantalum (Ta) coatings on carbon foams using the chemical vapor deposition (CVD) method to enhance their compressive strength. Two types of open-cell carbon foams, CF-1 and CF-2, with different strut diameters, were examined. The morphology and uniformity of the coatings were characterized, and the effect of coating thickness on the compressive strength of the foams was systematically analyzed. An empirical model was proposed and successfully validated, showing that the compressive strength is proportional to the coating thickness and the square of the strut diameter. The experimental results demonstrate that considering mass transfer and reaction kinetics can significantly improve the uniformity of coatings on larger substrates. Furthermore, the Ta coatings significantly increased the compressive strength of the foams, with the relationship between compressive strength, coating thickness, and strut diameter being in good agreement with the predictions of the proposed model. The study highlights the potential of tailored metallic coatings to enhance the mechanical properties of porous materials while maintaining their lightweight characteristics, emphasizing the importance of optimizing coating parameters for large-scale applications.

## Introduction

Open-cell foam materials exhibit superior properties, making them highly advantageous for numerous applications across diverse fields^1,2^. Characterized by their interconnected pore structure, these materials offer exceptional permeability, lightweight composition, high surface area, and effective thermal and acoustic insulation^3,4^. These unique attributes have led to their widespread use in biomedical engineering^5^, environmental protection^6^, energy storage^7^, and various industrial processes^8–10^.

In extreme high-temperature conditions, refractory metal foams and carbon foams are commonly used due to their ability to withstand severe thermal stress. Refractory metal foams, made from metals such as tungsten and molybdenum, exhibit excellent mechanical strength and thermal conductivity^11,12^. However, their high density and cost can be significant drawbacks. On the other hand, carbon foams are valued for their low density, high thermal resistance, and good electrical conductivity. Despite these advantages, carbon foams often suffer from brittleness and oxidation issues at elevated temperatures^13^. Both types of foams, while offering specific benefits, have limitations that can restrict their broader application in extreme environments.

To enhance the performance of carbon foams, various modification methods have been developed. These methods include fiber reinforcement to improve mechanical strength^14^, particle reinforcement for enhanced thermal and electrical properties^15,16^, chemical vapor deposition (CVD) or infiltration (CVI) for improved thermal stability and oxidation resistance^17,18^, and electroplating or electroless plating for increased electrical conductivity and corrosion resistance^19,20^. Among these, coating modifications stand out as particularly effective in significantly enhancing the properties of carbon foams for various applications. By applying protective coatings such as ceramics, metals, or polymers to the surface of carbon foams, their oxidation resistance, mechanical strength, and overall durability can be significantly improved. These coatings act as barriers, preventing the underlying carbon from reacting with the surrounding environment at high temperatures. This approach not only mitigates the brittleness and oxidation issues but also allows for the tailoring of surface properties to meet specific application requirements. The advantages of coating modifications make them a promising strategy for extending the applicability of carbon foams in harsh conditions.

Among the various coating materials, tantalum(Ta) coatings offer notable advantages. Ta is known for its exceptional high-temperature stability, making it an ideal choice for enhancing the thermal resistance of carbon foams^21^. The Ta coating provides a robust barrier against oxidation, significantly improving the material’s longevity under extreme conditions^22^. Additionally, Ta-coated carbon foams exhibit enhanced mechanical properties, including increased strength and toughness, which help to overcome the inherent brittleness of unmodified carbon foams. Furthermore, Ta’s excellent resistance to radiation damage makes it particularly suitable for applications in environments with high levels of radiation exposure, such as nuclear reactors and space missions^23^. These combined benefits of high-temperature resistance, improved mechanical performance, and radiation resistance make Ta-coated carbon foams a superior choice for advanced applications in challenging environments.

CVD offers significant advantages for depositing refractory metal coatings on porous foam materials. Firstly, CVD technology facilitates uniform and comprehensive coating coverage within intricate porous structures, demonstrating excellent permeability and compatibility with high melting point metals such as Ta and tungsten^24^. Secondly, the gas-phase reaction nature of CVD, conducted at elevated temperatures, enables the production of coatings with high purity and density. These coatings form robust interfacial bonds with the substrate, thereby enhancing adhesion and durability^25^. Additionally, CVD process parameters can be precisely controlled, allowing for the tailored adjustment of coating thickness and composition to meet specific application requirements^26,27^. Furthermore, refractory metal coatings produced via CVD exhibit exceptional high-temperature stability and corrosion resistance, ensuring the integrity and performance of porous foam materials in high-temperature and corrosive environments. This significantly augments the overall performance and application potential of these materials. However, for larger-sized substrates, the insufficient uniformity of the coating prepared by CVD is an important issue that requires attention.

This work employed CVD to fabricate Ta coatings on the surface of carbon foam. The basic structure and morphology of the coatings were examined, and a brief analysis of coating uniformity was conducted. The compressive performance of carbon foam with different strut diameters after depositing various thicknesses of Ta coatings was compared. An empirical formula predicting the enhancement effect of coatings on the compressive performance of porous materials was proposed and validated. This study differentiates itself from previous works by examining the combined influence of coating thickness and strut diameter on the mechanical properties of carbon foams. While previous studies have explored similarly coated foams under compression, they typically focused on single factors, such as coating material or foam structure^28,29^, without considering the combined impact of these factors on overall strength and durability. Moreover, this work emphasizes the uniformity of the deposition process, which is often overlooked in other studies. The uniform distribution of the Ta coating is critical to achieving consistent mechanical properties across the foam, significantly when scaling up the deposition process for larger substrates.

## Experimental and materials

### Carbon foam sample

#### Sample preparation

**Fig. 1:**
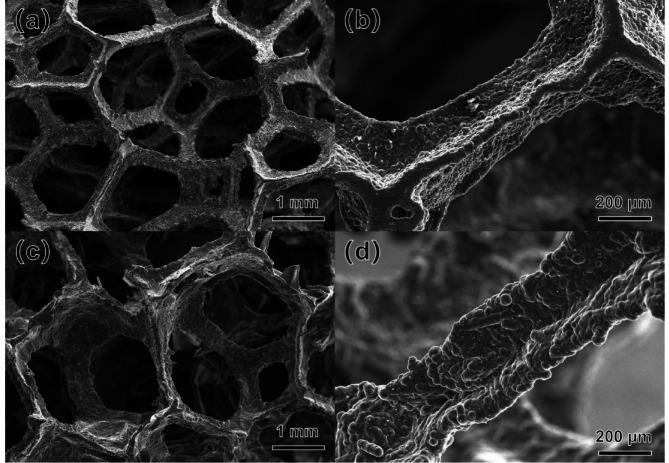
The morphology of CF-1 and CF-2 (**a**) CF-1;(**b**) CF-2.

The porous carbon foam used in this experiment was provided by Qingdao Gaotai New Materials Co., Ltd. Two types of carbon foam were used in the experiment, with their basic parameters shown in Table 1. Their porosity and apparent density are different, but the material, pore morphology, and pore density are consistent. Figure 1 shows the morphology of CF-1 and CF-2, their frameworks share the same morphology.

**Table 1:** Physical structural parameters of carbon foam.

Type	Apparent density (g/cm^3^)	True density (g/cm^3^)	Porosity	Pore density (PPI)
CF-1	0.031±0.002	1.44±0.04	97.8%	25
CF-2	0.094±0.003	1.42±0.03	93.4%	25

The metal Ta utilized was in the form of pure Ta sheets (purity > 99.95%), annealed through powder metallurgy. The reactive gases were chlorine and hydrogen, both subjected to drying and purification processes.

**Fig. 2:**
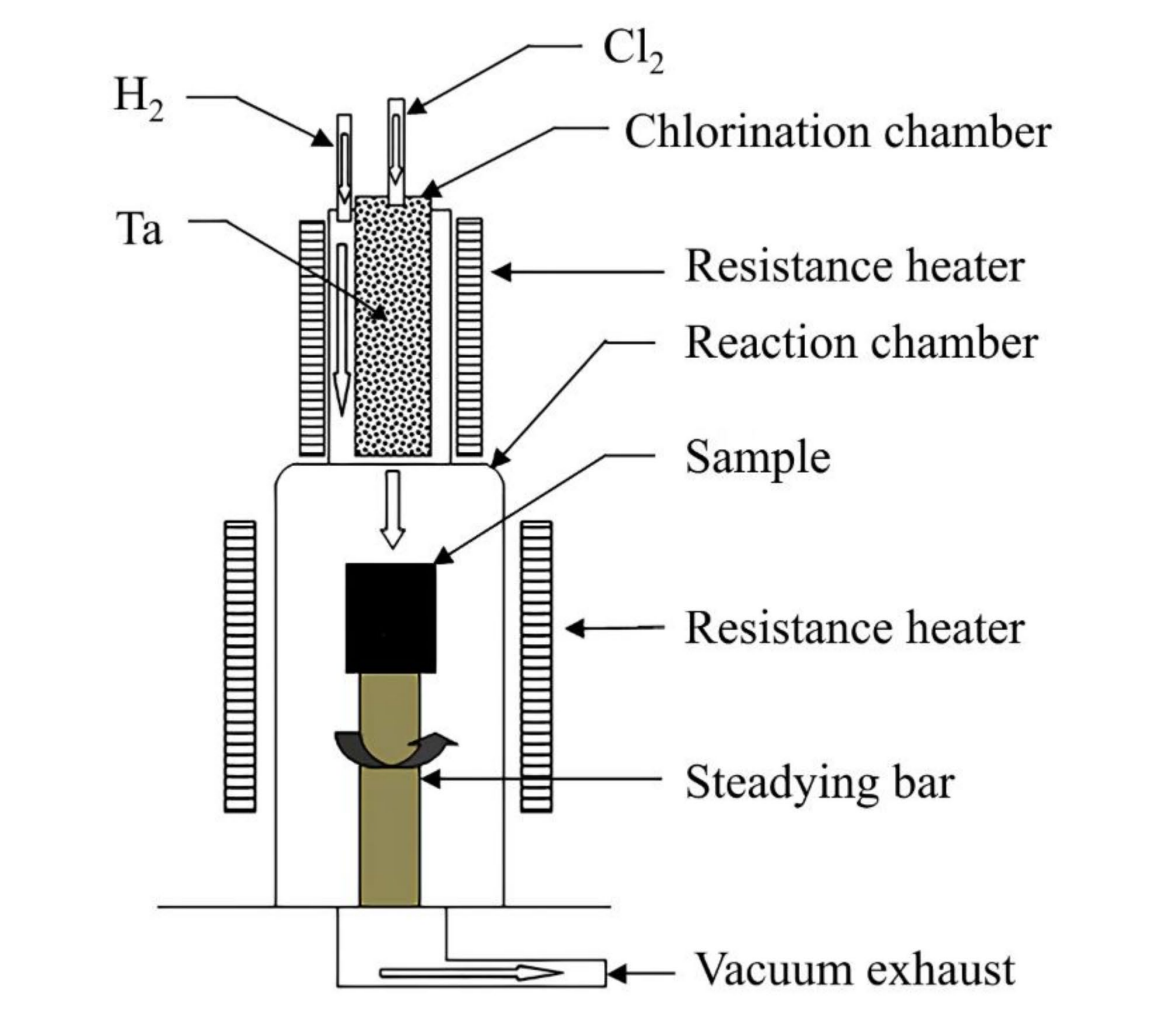
Schematic diagram of CVD experimental setup.

A custom-designed chlorination-reduction reaction apparatus was used for the Ta deposition experiments. A schematic of the reaction apparatus is shown in Fig. 2.

In the experiment, a rotor flowmeter was used to control the flow of the reaction gases. The chlorination chamber was heated to the chlorination temperature using an electric resistance furnace, and the temperature was measured with a platinum-rhodium thermocouple. The heating was conducted through wall heating.

In the experiment, the parameters for Ta chlorination, such as chlorination temperature, chlorine gas flow, hydrogen gas flow, and deposition temperature, were selected based on empirical values from preliminary studies. To ensure uniform distribution of Ta on the foam carbon framework, the deposition was carried out twice with the sample placement directions reversed. The specific deposition conditions are listed in Table 2.

**Table 2:** Experimental parameters.

Deposition temperature (°C)	Chlorination chamber temperature (°C)	Pressure (MPa)	Cl₂ gas flow rate (mL/min)	H₂ gas flow rate (mL/min)	Deposition time (hours)
1150	500	0.08	100	1000	2

The fundamental process of depositing Ta on porous carbon foam using CVD involves initially a chlorination reaction between chlorine gas and Ta within the chlorination chamber, resulting in the formation of TaCl5 gas. Subsequently, this TaCl5 gas, upon reaching the deposition chamber and interacting with the surface of the porous carbon foam heated to a specific temperature, reacts with hydrogen gas. This reaction reduces the Ta, facilitating its deposition onto the surface of the porous carbon foam’s framework. The primary steps are as follows:

1. Initially, the porous carbon foam samples were ultrasonically cleaned with alcohol to remove impurities from the surface and pores. Subsequently, the samples were dried using dry nitrogen gas before being placed in the deposition chamber.
2. The inlet and outlet gas pipes were connected, and the entire system was inspected for airtightness. The system was then heated under vacuum conditions.
3. When the chlorination temperature reached 400 °C and the substrate temperature rose to 1150 °C, the flow rates of chlorine and hydrogen gases were sequentially adjusted, and the deposition process was initiated.
4. After 2 h of deposition, the chlorine and hydrogen gases were shut off, and the system was cooled under vacuum conditions.
5. When the temperature dropped below 100 °C, the reaction apparatus was opened to collect samples. The chemical reaction equation during the deposition process is as follows:

1 $$2{\text{Ta}}({\text{s}}) + 5~{\text{Cl}}_{2} ({\text{g}}) \to 2{\text{TaCl}}_{5} ({\text{g}})$$

2 $${\text{2TaC}}{{\text{l}}_{\text{5}}}({\text{g}})\,+\,{\text{5}}{{\text{H}}_{\text{2}}}({\text{g}}) \to {\text{2Ta}}({\text{s}})\,+\,{\text{1}}0{\text{HCl}}({\text{g}})$$

### **Basic characterization**

The microstructure of the surface and cross sections of the foams were characterized by field emission scanning electron microscopy (SEM). X-ray diffraction (XRD) analysis determined the samples’ phase composition.

### Deposition uniformity

**Fig. 3:**
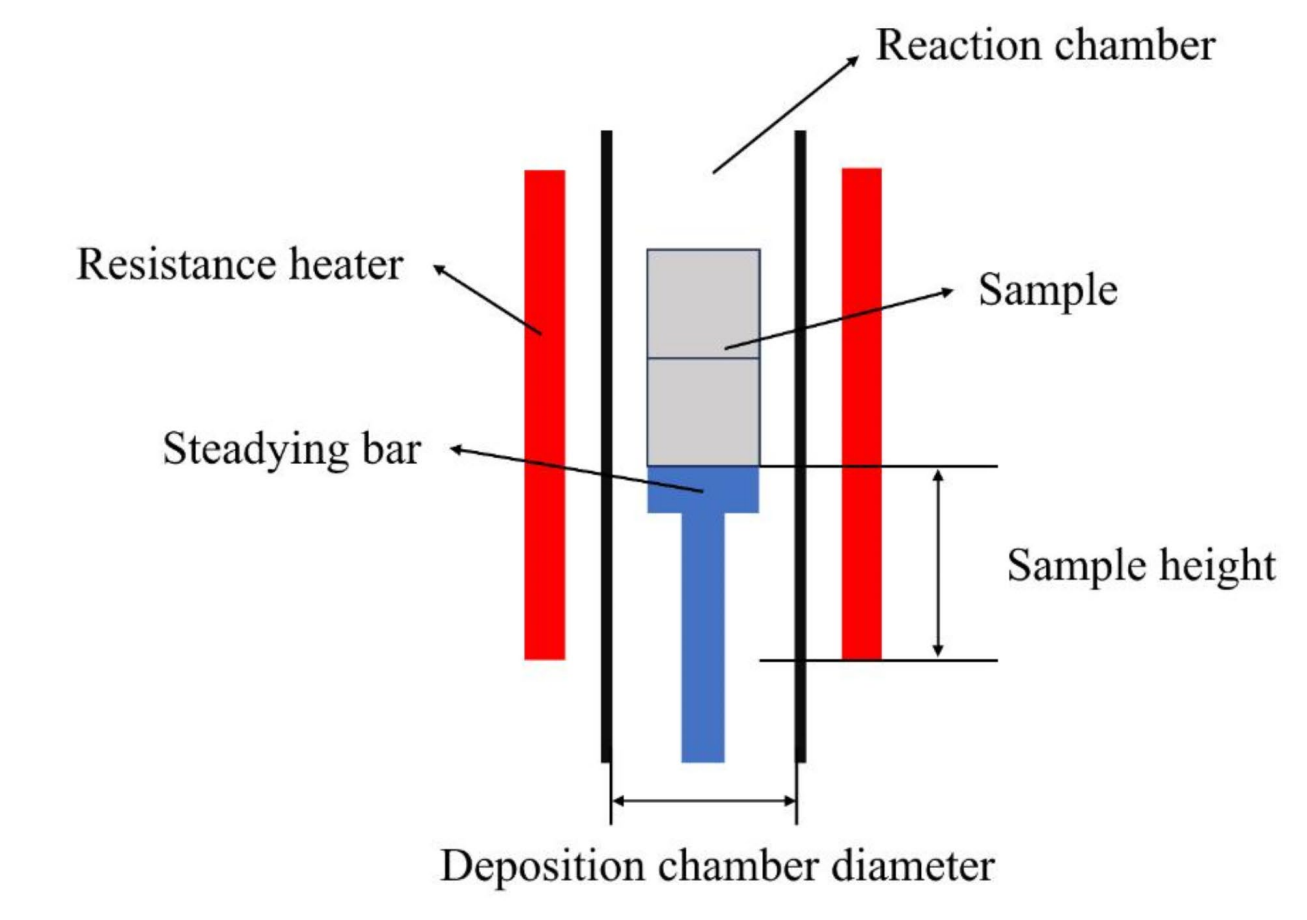
Schematic diagram of the sample positioning within the deposition chamber.

In the experiment, a giant foam carbon substrate with dimensions of Φ60 mm × 50 mm was placed at different heights and in deposition chambers with varying diameters within a vertical tube furnace for deposition, as shown in Fig. 3. A similarly sized porous material was placed below the sample as a base to minimize mass transfer interference caused by the base.

**Fig. 4:**
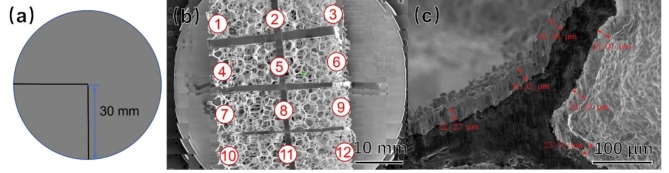
Schematic Diagram of Data Sources for Uniformity Analysis.

The deposited samples were cut, as shown in Fig. 4. The samples were then observed using a SEM, and the coating thickness was recorded at 12 locations. The coating thickness perpendicular to the measurement plane was measured at each location, and an average of six measurements was taken.

### Quasi-static compression tests

#### Test parameters and conditions

Mechanical performance tests were conducted at a constant compression rate of 0.002 mm/s(strain rate was 2 × 10^−4^ s^−1^). Each specimen was a cylindrical sample with dimensions of Φ20 mm × 10 mm. Due to the unique porous structure, specific guidelines must be followed for compression experiments. The most critical parameters are sample size and load transfer^30^. To avoid the influence of the Weibull size effect, all samples should be cut to the same size. The stability of test results for materials with large pores is related to the ratio of pore size to sample size. Considering sample uniformity and size effects, the sample size was set to a cylinder with dimensions of Φ20 mm × 10 mm. Additionally, for porous materials, the sides of the sample are free, while the ends of the sample are in contact with the loading plates. The cells near the free surface experience less constraint than those in the bulk, resulting in a more minor contribution to overall stiffness and strength. Therefore, samples were cut using an electrical discharge wire cutting machine, and the cut surfaces were polished to ensure smoothness. This preparation method helps minimize artifacts that could affect the mechanical testing results.

#### Compression properties

When evaluating mechanical properties, the following properties were determined from the stress-strain curve: elastic modulus (E), compressive strength ($\:{\sigma\:}_{C}$), energy absorption capacity at the densification point (W), and energy absorption efficiency (e). The elastic modulus was obtained using the linear fitting tool provided by Origin 2020 software. The compressive strength $\:{\sigma\:}_{C}$ was taken as the first peak stress after the elastic stage. The energy absorption capacity and energy absorption efficiency were calculated using Eq. 3 and Eq. 4, respectively.

3 $$\:W={\int\:}_{0}^{{\epsilon\:}_{D}}{\sigma\:}_{\epsilon\:}d\epsilon\:$$

4 $$\:e=\frac{{\int\:}_{0}^{{\epsilon\:}_{D}}{\sigma\:}_{\epsilon\:}d\epsilon\:}{{\sigma\:}_{C}{\epsilon\:}_{D}}$$

Where $\:{\epsilon\:}_{D}$ represents the densification strain, defined as the strain at the onset of densification. However, since there is usually no abrupt point marking the beginning of the densification stage, it is conveniently set to 60% in this study for calculation purposes.

## Result and discussion

### Morphology and composition of the composite

**Fig. 5:**
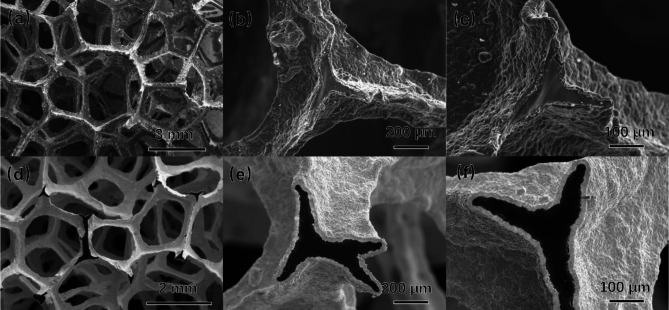
Surface morphology of porous carbon foams before and after Ta deposition. (a), (b), (c) Before deposition; (d), (e), (f) After deposition.

Figure 5 shows the morphologies of carbon foams at different magnifications before and after Ta deposition. The polyhedral morphology of the porous foam carbon, characterized by large openings and columnar frameworks, was maintained post-Ta deposition. Consistent porous structures with stable pore sizes and interconnected porosity in the foam carbon were observed in both pre and post-Ta deposition. It is inferred from these observations that CVD technology enables the effective deposition of metallic Ta onto the porous foam carbon framework. This technique maintains the structural integrity and porosity of the foam carbon, thereby preserving its essential characteristics as a porous material.

As observed in Fig. 5a, b, c, prior to Ta deposition, the surface of the original porous foam carbon’s framework was uneven, with irregular sizes. These defects were highly detrimental to the mechanical properties of the porous foam carbon. After Ta deposition, the metal Ta essentially enveloped the framework of the porous foam carbon, resulting in a smoother, more uniform structure in terms of shape and size, as shown in Fig. 5d, e, f. Furthermore, during the deposition process, Ta penetrated the pores and defects of the framework. This interaction not only enhanced the adhesion between the Ta coating and the porous foam carbon, strengthening their bond, but also reduced or eliminated defects in the foam carbon framework, thereby improving its mechanical properties.

**Fig. 6:**
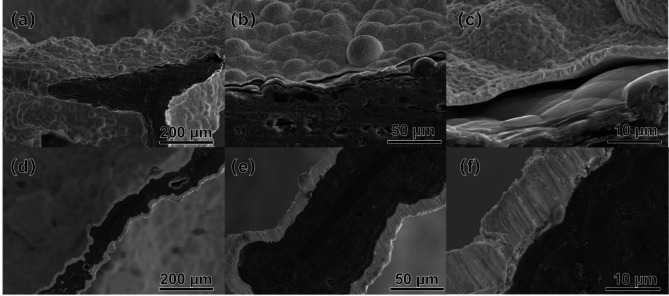
The microstructure of the composite foam interface. (**a**), (**b**), (**c**) Thin coating; (**d**), (**e**), (**f**) Thicker coating.

Figure 6 shows the microstructure of the composite foam interface. It can be observed that when the coating is thin, it is more prone to separation from the substrate, whereas a thicker coating adheres better to the substrate. This can be attributed to the stress concentration caused by the difference in the thermal expansion coefficients between the substrate and the coating. The thin coating cannot relieve these stresses, leading to the separation between the coating and the substrate. Additionally, it can be seen that the coating provides relatively complete coverage of the substrate surface, which allows for better mechanical bonding between the coating and substrate when the coating is thicker.

**Fig. 7:**
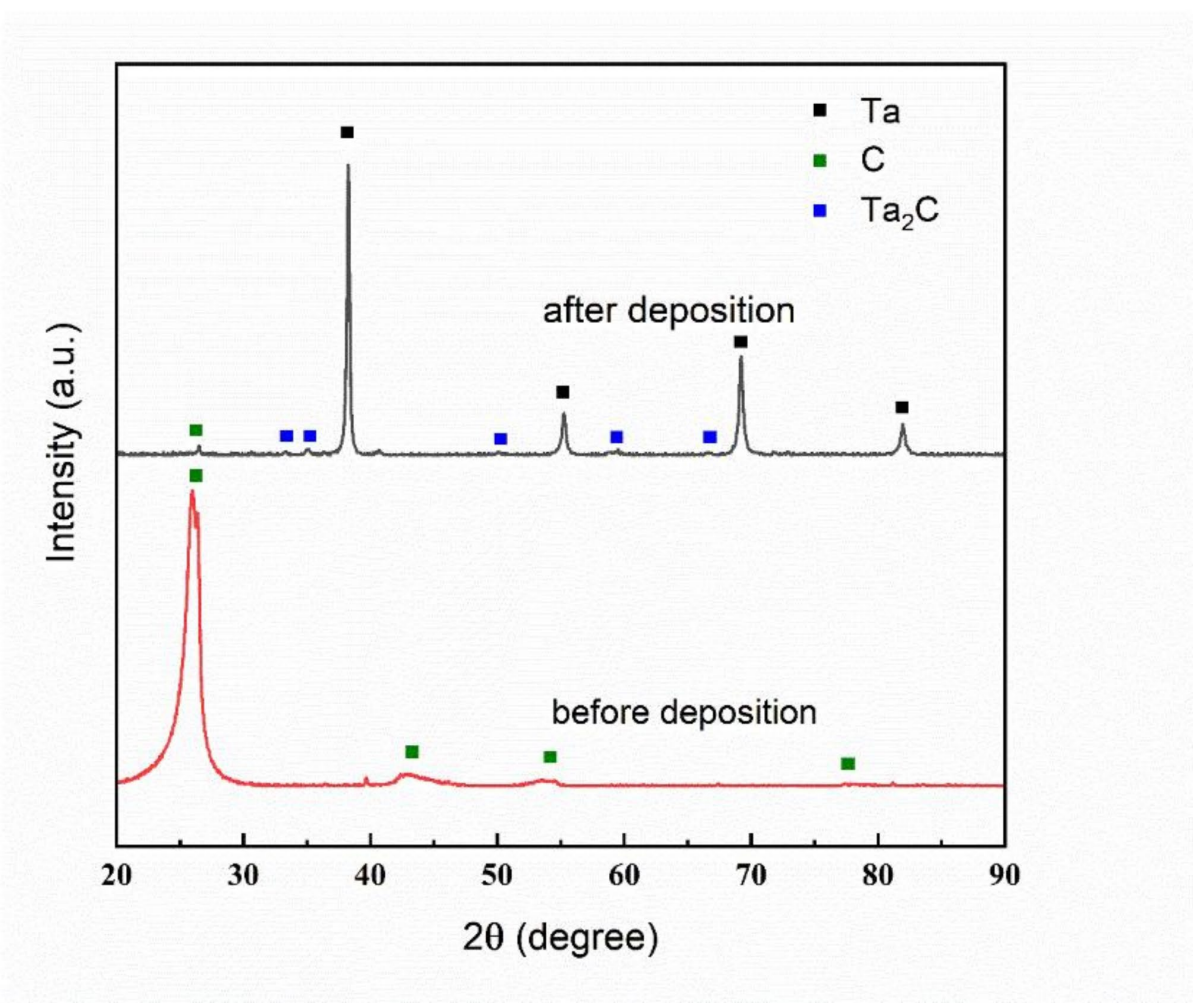
Results of XRD analysis before and after deposition.

**Fig. 8:**
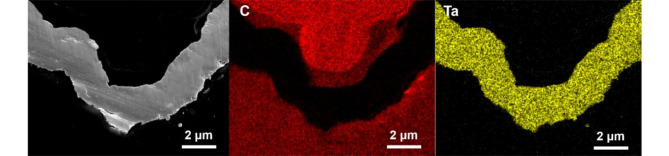
EDS analysis of the coating cross-section.

Figure 7 presents the XRD results before and after deposition. Figure 8 shows the EDS analysis results of the coating cross-section. The XRD and EDS analysis confirmed the successful deposition of a Ta coating with a body-centered cubic (BCC) structure, corresponding to the $\:\alpha\:$-phase of Ta. No $\:\beta\:$-phase peaks were detected, indicating effective suppression of the metastable $\:\beta\:$-phase under the applied deposition conditions. The average crystallite size of the Ta coating, calculated using the Scherrer equation, was approximately 30 nm. Additionally, the presence of minor Ta₂C peaks suggests partial interaction between the Ta coating and the glassy carbon substrate during the CVD process. The observed $\:\alpha\:$-phase, with its superior ductility and conductivity, coupled with the nanocrystalline structure, is expected to enhance the mechanical properties of the coating^31^, demonstrating the efficacy of the applied deposition parameters for high-performance applications. Compared to electrochemical deposition^32,33^, which introduces various impurities, the CVD method results in fewer impurities and produces higher-quality coatings.

Since the deposition thickness is relatively small compared to the thickness of the substrate framework, the deposition thickness can be calculated using Eq. 5.

5 $$\:d=\frac{\varDelta\:m}{{\rho\:}_{c}A}$$

Where *d* represents the deposition thickness, $\:{\rho\:}_{c}$ is the coating density, and *A* denotes the surface area of the substrate. The sample apparent density after deposition, $\:{\rho\:}_{a}$​ is calculated using Eq. 6.

6 $$\:{\rho\:}_{a}={\rho\:}_{b}+\frac{\varDelta\:m}{V}$$

**Fig. 9:**
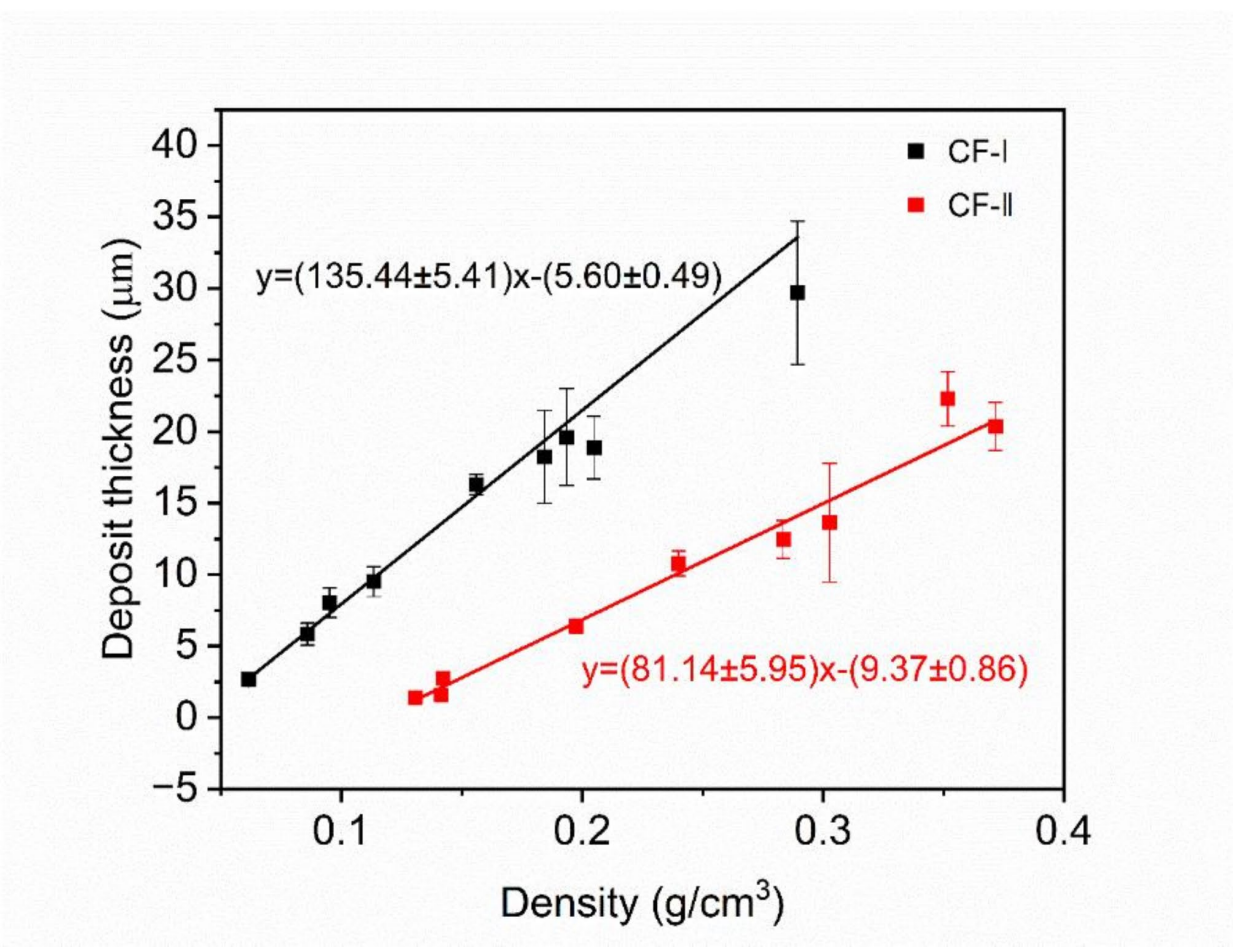
The relationship between deposition thickness and final density. The solid line represents the least-squares fitting of the data.

As shown in Table 1, the apparent density of CF-2 is three times that of CF-1, while their true densities are the same. This indicates that the framework volume of CF-2 is three times that of CF-1. Treating the framework as a cylindrical body, with the height remaining constant, a tripling of volume implies that the surface area would increase by a factor of $\:\sqrt{3}$​. Consequently, in the d-$\:{\rho\:}_{a}$ plot, the slope for CF-1 should be $\:\sqrt{3}$​ times that of CF-2. Figure 9 presents the experimental d-$\:{\rho\:}_{a}$​ plot. The relationship between d and $\:{\rho\:}_{a}$ was obtained using least-squares fitting. The results show that the ratio $\:\frac{{k}_{1}}{{k}_{2}}\approx\:1.67$, which aligns well with the theoretical analysis.

### Deposition uniformity

In CVD, deposition uniformity is a critical consideration for large substrates. The deposition rate is mainly influenced by two factors^34^: the kinetics of the chemical reaction and the transport of reactants and products in the gas phase.

The changes in the mass of the obtained samples are shown in Table 3. It can be seen that there is a strong correlation between the deposition amount and height. Generally, the higher the height, the greater the deposition amount. However, there is a plateau at a certain height range where changes in height have less impact on the deposition amount. Additionally, the size of the deposition chamber significantly affects the results. The deposition amount of Ta in a 100 mm chamber is less than half of that in an 80 mm chamber. This can be attributed to the fact that, with the same flow rate of reactant gases, the amount of reactants passing through the samples decreases in larger reactors.

**Table 3:** Basic information of experimental results.

Serial number	Deposition chamber diameter (mm)	Sample height (cm)	Amount of Ta chloride (g)	Mass increase (g)
1	80	10.5	67	19.68
2		8.5	84	15.98
3		6.5	63	16.62
4		4.5	68	9.02
5	100	10.5	79	7.40

**Fig. 10:**
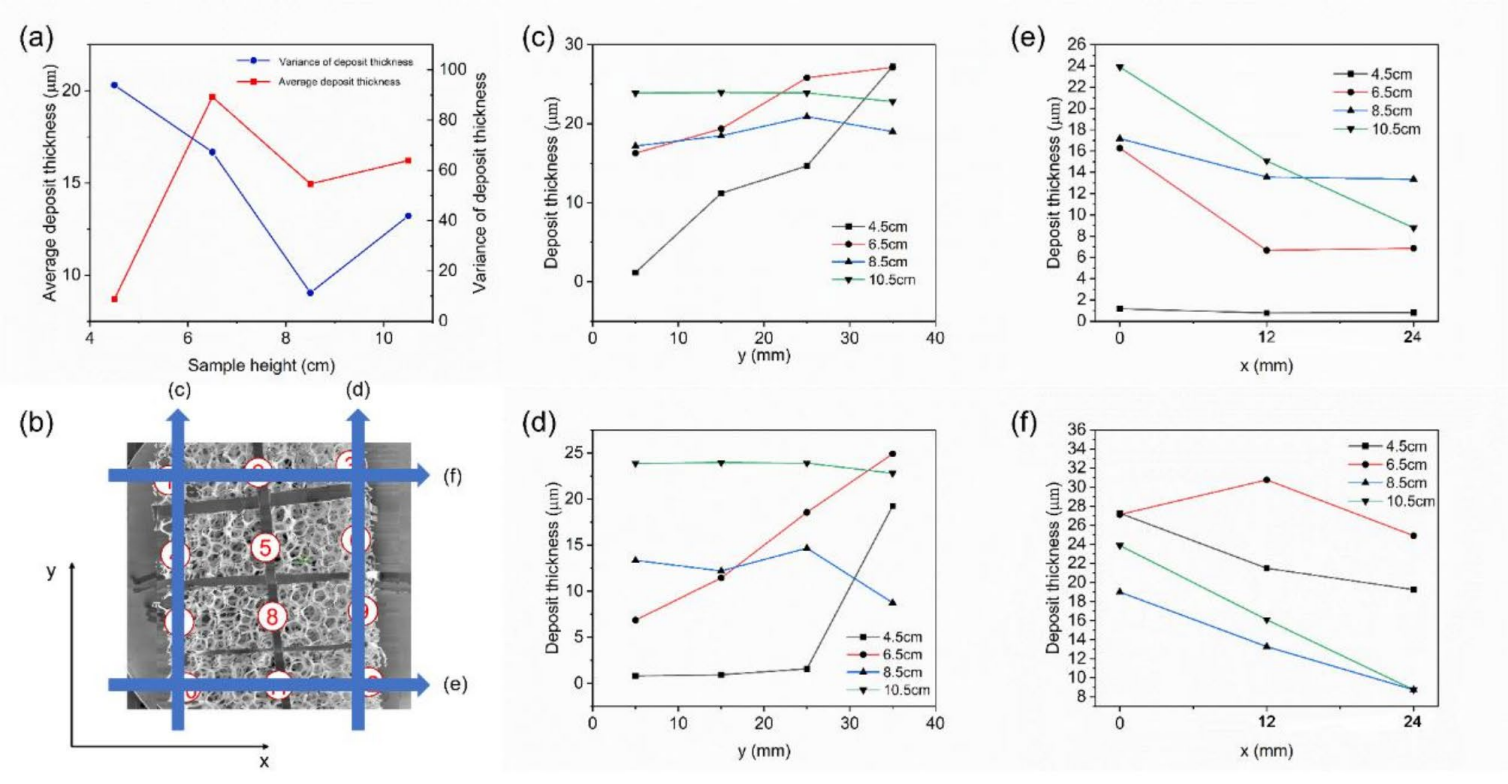
(**a**) Deposition thickness and thickness variance of samples at different positions in the deposition chamber; (**b**) Schematic diagram of data sources for (**c**), (**d**), (**e**), (**f**); (**c**), (**d**), (**e**), (**f**) Deposition thickness in different directions within various samples.

The results are shown in Fig. 10. Samples placed at higher positions exhibited better axial uniformity. Due to the use of a hot-wall reactor, deposition also occurred on the walls of the deposition chamber. For samples placed at lower positions, a large amount of reactants was consumed on the chamber walls, reducing the concentration of reactants and resulting in poor axial uniformity. However, samples at lower positions had better radial uniformity, primarily due to mass transfer effects.

Figure 2 shows a schematic of the connection between the chlorination chamber and the deposition chamber. Hydrogen enters the deposition chamber from the outside, while Ta pentachloride enters the chlorination chamber from the center. The mixing of hydrogen and Ta pentachloride is less effective at higher positions, resulting in poorer axial uniformity. Overall, the CVD method can achieve good uniformity in coating deposition on complex surfaces, offering a significant advantage over the physical vapor deposition (PVD) method.

### Quasi-static compression tests

**Fig. 11:**
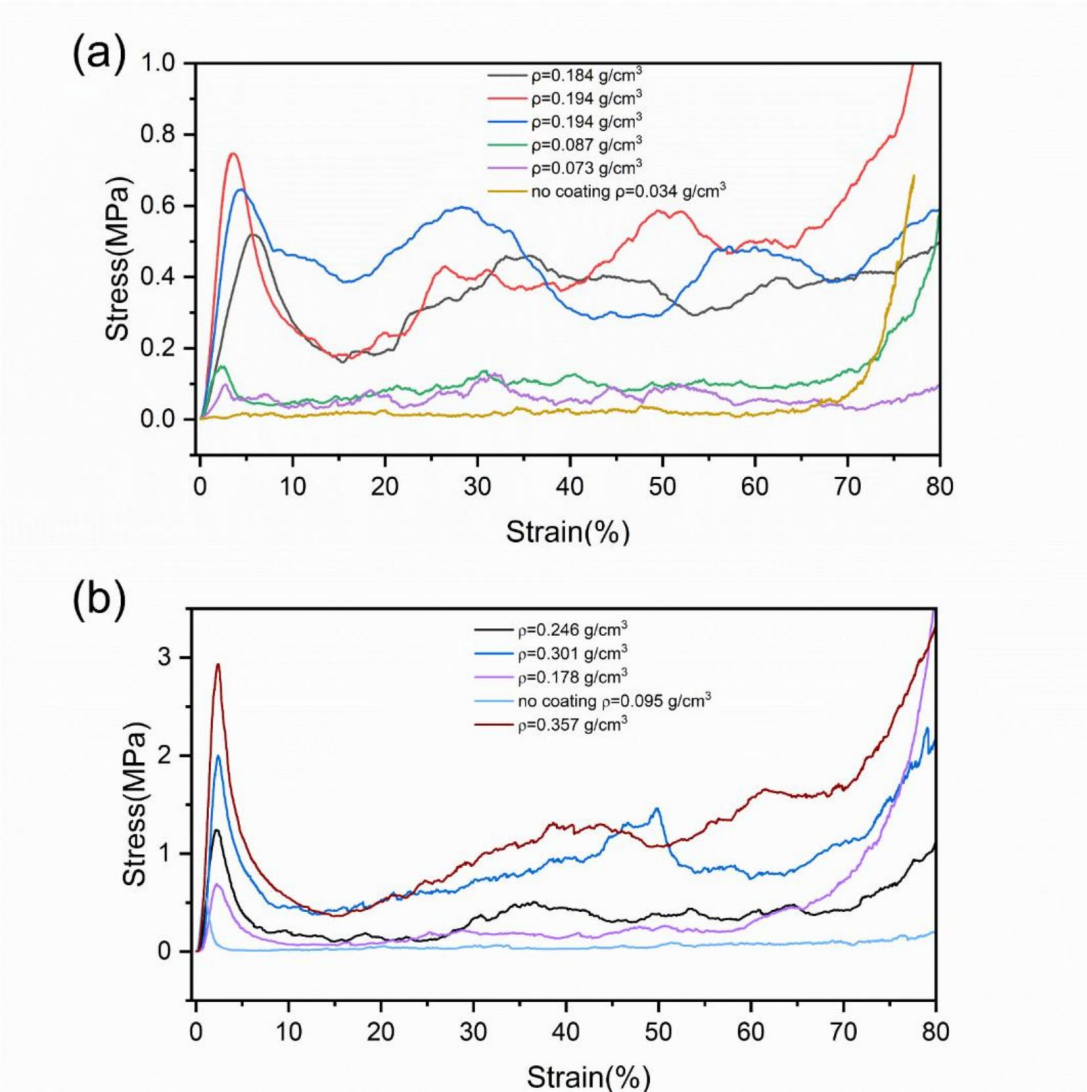
Quasi-static compression test stress-strain curve: (**a**) CF-1; (**b**) CF-2.

Figure 11 shows the quasi-static compression stress-strain curves of two different carbon foam structures: standard and Ta/C composite foams. The curves can be roughly divided into three regions: the linear region, the plateau region (plastic collapse region or brittle fracture region), and the densification region. In the linear region, the structure primarily undergoes recoverable bending deformation, and the elastic modulus is a critical parameter at this stage. After reaching the peak stress, the stress drops rapidly. At this point, the cell edges undergo plastic yielding, buckling, or fracture. These three potential failure mechanisms compete with each other, with the mechanism requiring the lowest stress dominating.

Table 4 shows the results obtained from the stress-strain curve analysis. The compressive modulus and compressive strength of carbon foam significantly increase after Ta deposition. Even the samples with the thinnest Ta deposition exhibited more than double the compressive strength. The shape of the curve is typical of open-cell foamed materials’ stress-strain behavior. Analyzing the compression stress-strain curve of open-cell foam materials without coating reveals a rapid stress drop after reaching the elastic limit, maintaining a low-level plateau region. This behavior is attributed to the structure of open-cell foam materials, which consist of ligaments and struts without cell walls. The primary failure mechanism is the bending and fracture of the struts. When the material reaches its elastic limit, the struts begin to buckle or fracture, leading to a loss of load-bearing capacity and the formation of a stress plateau. Since carbon foam is a brittle material, these structural elements absorb most of the energy during buckling and fracture, causing the stress to remain relatively constant at a low level over a wide range of deformation.

In the plateau region, the stress-strain curve exhibits significant fluctuations. This can be attributed to the material’s brittleness and the effect of the sample size. The performance of the plateau region in the stress-strain curve of porous materials is primarily related to two factors: the material’s plasticity and brittleness, as well as the porosity of the sample. In the plateau region, the energy absorbed per unit volume of compression is mainly provided by the bending deformation energy and fracture energy of the supporting struts. When the sample’s size is small relative to the pore size, meaning there are fewer supporting struts, the failure of a small number of struts can cause significant fluctuations in the stress in the plateau region, and the effects of defects and randomness are more pronounced. Additionally, for brittle materials, the failure stress of individual struts varies greatly, whereas for ductile materials, the stress variation is more gradual.

**Table 4:** Basic information of experimental results.

	Slope of the elastic region	Maximum stress (MPa)	Density (g/cm^3^)	Energy absorption	Energy absorption efficiency
CF-1	0.116	0.52	0.184	19.7	0.63
	0.313	0.75	0.194	23.6	0.52
	0.222	0.64	0.193	25.9	0.67
	0.098	0.15	0.087	5.2	0.60
	0.038	0.10	0.073	3.8	0.64
	0.001	0.03	0.061	1.0	0.56
	0.142	0.41	0.154	16.2	0.65
	0.188	0.32	0.113	6.0	0.31
CF-2	0.959	1.23	0.246	19.2	0.26
	1.358	1.83	0.344	43.7	0.39
	1.674	1.99	0.303	47.9	0.40
	0.610	0.68	0.178	11.5	0.28
	0.913	0.54	0.097	2.9	0.09
	2.057	2.92	0.357	60.4	0.34
	2.613	2.99	0.367	62	0.35

**Fig. 12:**
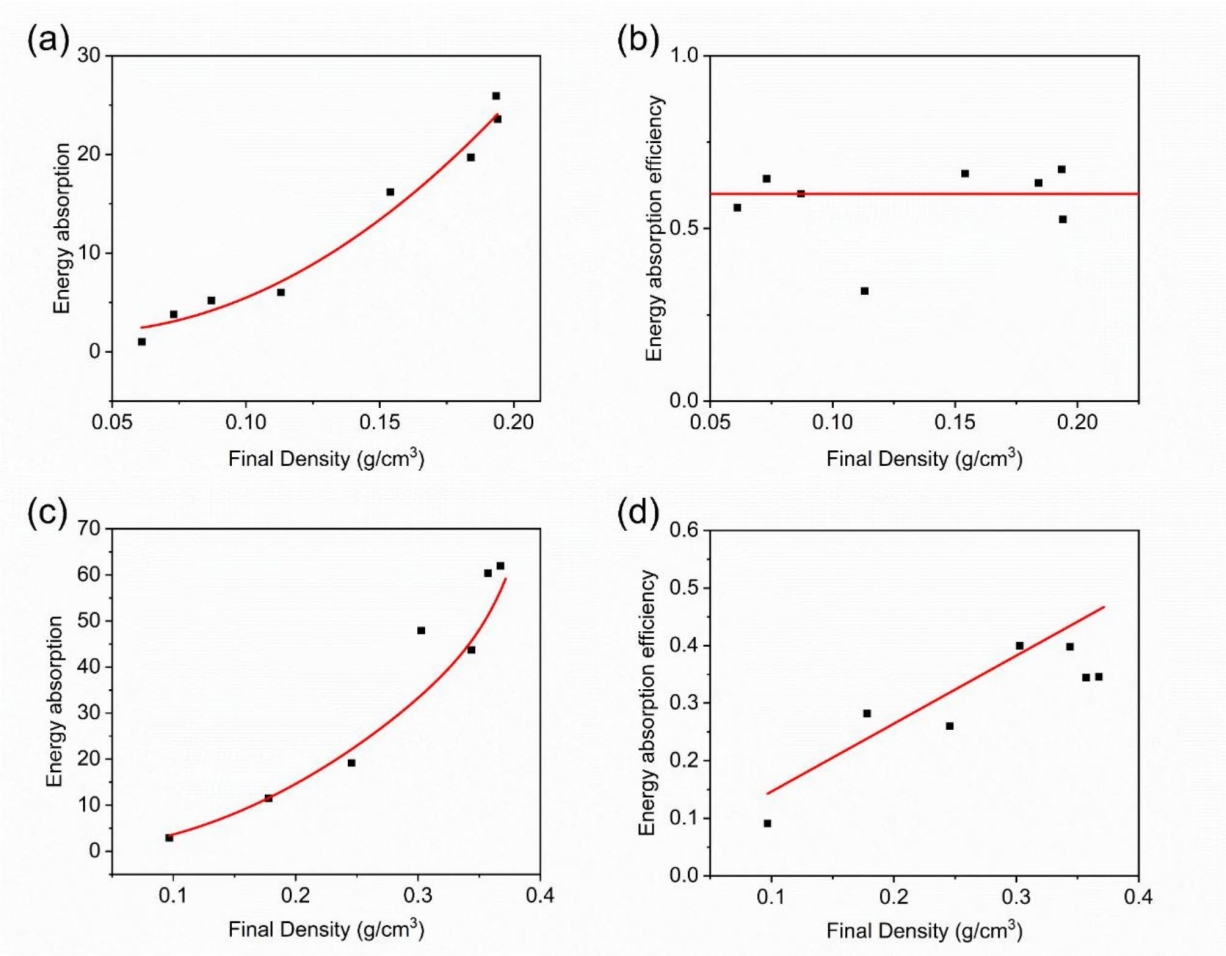
Energy Absorption Capacity/Efficiency-Density Curve: (**a**), (**b**) CF-1; (**c**), (**d**) CF-2. The red line represents the trendline.

Figure 12 shows the relationship between the energy absorption capacity and density of carbon foam after Ta deposition. It can be seen that the energy absorption capacity significantly increases with the thickening of the coating. The energy absorption efficiency of CF-1 remains relatively stable, while the energy absorption efficiency of CF-2 increases as the coating thickness increases.

For the smaller framework of CF-1, the relatively thick Ta coating dominates the material’s mechanical behavior, allowing it to undergo significant deformation under compressive loads without immediate fracture. This plastic deformation mechanism enables the material to absorb more energy after reaching the elastic limit, resulting in a smoother stress decline curve with multiple small stress fluctuations.

In contrast, for the larger framework of CF-2, the Ta coating is relatively thinner compared to the framework, and the mechanical properties of the framework dominate the material. Under high stress, brittle fracture is more likely to occur, leading to a sharp stress drop. The thinner coating is insufficient to significantly enhance the overall plastic deformation capacity, resulting in lower energy absorption efficiency. However, as the coating thickness increases, its influence on the material’s mechanical properties strengthens, and the deformation mechanism gradually shifts toward plastic deformation, significantly improving energy absorption efficiency.

Based on the cubic unit cell model proposed by Gibson and Ashby^35^, this paper establishes an empirical model to predict the enhancement of foam compressive strength by coatings. In the cubic unit cell model, the deformation of struts in open-cell foam is predominantly bending deformation.

In the cubic unit cell model, the plastic collapse strength $\:{\sigma\:}_{C}$ of the open-cell foam is related to the fully plastic bending moment $\:{M}_{P}$ of the foam struts as follows^35^:

7 $$\:{\sigma\:}_{C}\propto\:\frac{{M}_{P}}{{L}^{3}}$$

Where $\:L$ is the strut length. It can be seen from the formula that the macroscopic compressive strength is proportional to the plastic limit bending moment $\:{M}_{P}$ of the struts. Assuming the mechanical behavior of the struts is ideally elastic-plastic, $\:{M}_{P}$​ can be expressed as:

8 $$\:{M}_{P}=\int\:{S}_{y}ydA$$

Where $\:{S}_{y}$ is the yield strength of the material, and $\:y$ is the distance to the central axis. By calculating the stress at each point (or element) on the beam’s cross-section under bending and combining it with their distance from the neutral axis, the contribution of the entire cross-section to the bending moment is obtained, ultimately leading to the determination of the beam’s full plastic moment. Using mechanical principles, Eq. 8 can be extended to composite materials reinforced with coatings^36^. $\:{M}_{P}$​ consists of two parts: the skeleton and the coating.

9 $$\:{M}_{P}=\underset{{A}_{C}}{\overset{\:}{\int\:}}{S}_{y}^{c}ydA+\underset{{A}_{Ta}}{\overset{\:}{\int\:}}{S}_{y}^{Ta}ydA$$

Since the matrix exhibits brittle fracture and has a high porosity, its compressive strength is greatly affected by defects. This paper mainly considers the reinforcing effect of the coating on the matrix. Given that the coating thickness $\:d$ is small relative to the average diameter of the skeleton, Eq. 9 can be simplified to:

10 $$\:{M}_{P}^{Ta}={\int\:}_{0}^{2\pi\:}{S}_{y}^{Ta}y\left(\theta\:\right)d\sqrt{{y}^{2}\left(\theta\:\right)+{{y}^{{\prime\:}}}^{2}\left(\theta\:\right)}={C}_{1}{S}_{y}^{Ta}d{y}_{0}^{2}$$

Where $\:y\left(\theta\:\right)$ is the polar coordinate function of the strut cross-sectional edge, $\:{y}_{0}$​ is the average radius of the strut, and $\:{C}_{1}$​ is a proportional constant. Thus, the reinforcing effect of the coating on the carbon foam can be expressed as:

11 $$\:\varDelta\:\sigma\:={C}_{2}\frac{{M}_{P}^{Ta}}{{L}^{3}}={C}_{1}{C}_{2}{S}_{y}^{Ta}\frac{d{y}_{0}^{2}}{{L}^{3}}={C}_{3}d{y}_{0}^{2}$$

From Eq. 11, it can be seen that when the coating is thin, the enhancement of compressive strength is proportional to the coating thickness and proportional to the square of the average radius of the struts.

**Fig. 13:**
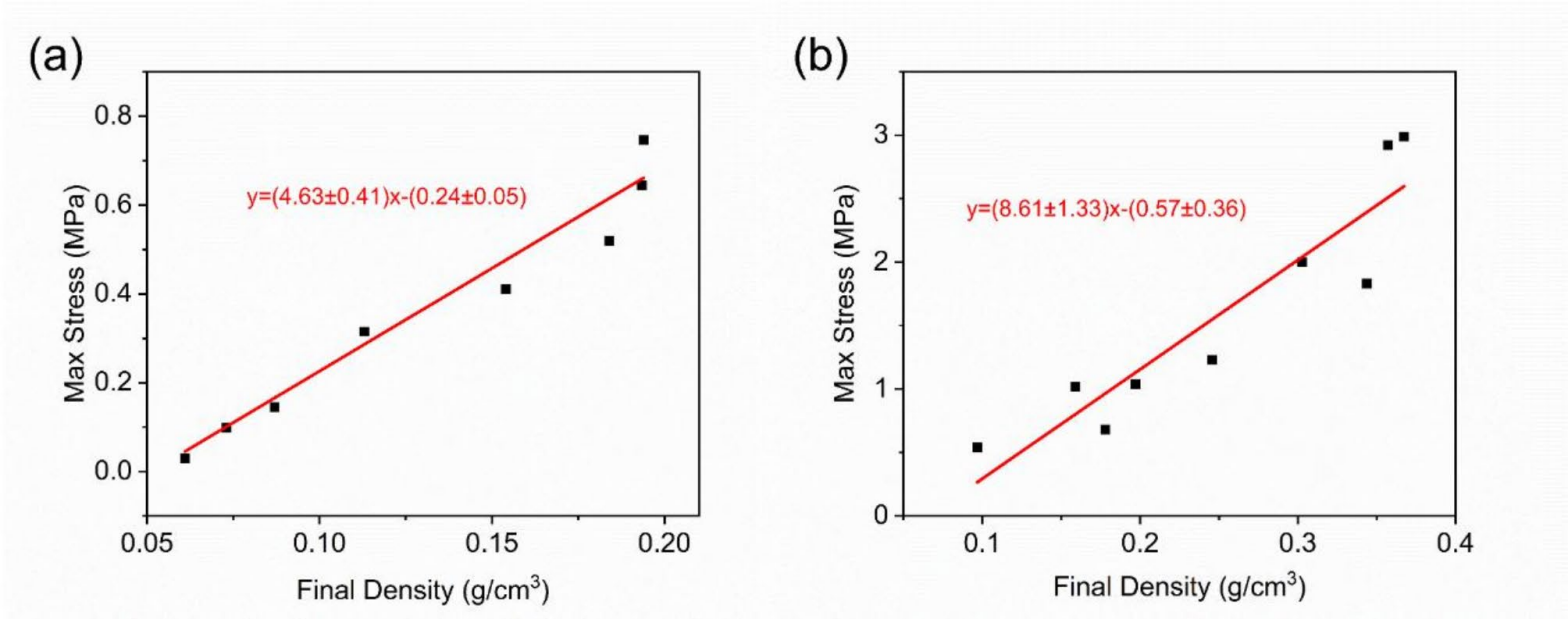
Maximum Stress-Density Relationship Diagram: (**a**) CF-1; (**b**) CF-2.

Figure 13 shows the compressive strength-density relationship of CF-1 and CF-2 carbon foams after Ta deposition. Using the least squares method for fitting, the results are CF-1 $\:{R}^{2}=0.96$ and CF-2 $\:{R}^{2}=0.87$, indicating a good linear correlation between compressive strength and density.

Based on the results of Fig. 9, compressive strength is linearly correlated with deposition thickness. The average strut radius of CF-2 is $\:\sqrt{3}$ times that of CF-1. Therefore, in the compressive strength-density relationship graph, the slope of CF-2 should be $\:\sqrt{3}$ times that of CF-1. The results show that $\:\frac{{k}_{2}}{{k}_{1}}\approx\:1.86$, which agrees well with the model’s prediction.

## Conclusion

In this study, Ta coatings with an average grain size of approximately 30 nm in the $\:\alpha\:$-phase, were successfully deposited on two types of open-cell carbon foams (CF-1 and CF-2) using CVD method. The results demonstrated that both the coating thickness and the strut diameter significantly influenced the compressive strength of the foams. A positive correlation was observed between coating thickness and compressive strength, with the compressive strength being proportional to the square of the strut diameter. Additionally, a linear relationship was found between the deposition thickness and the final density in the case of thinner coatings.

The uniformity of the coatings at different heights of the substrate was analyzed. It was found that optimizing deposition parameters to reduce gas loss and achieve a more uniform distribution within the substrate could significantly improve the coating uniformity. For larger-sized carbon foams, adjusting deposition parameters was found to be particularly important for enhancing coating uniformity.

The Ta coatings significantly increased the compressive strength of the carbon foams. When the coating thickness relative to the supporting struts was larger, the material’s formability increased. Conversely, thinner coatings made the material more prone to brittleness, similar to the behavior of the substrate. The maximum stress of the composite material was found to be proportional to the square of the foam strut diameter and the coating thickness.

Nevertheless, in the context of scaling up the CVD process for industrial applications, energy consumption emerges as a major cost factor. The high temperatures and vacuum conditions required for the deposition process, especially when applied to larger-sized foams, demand substantial energy input. As such, further research should focus on optimizing energy efficiency within the CVD process to reduce production costs and improve the economic feasibility of large-scale manufacturing. This optimization will also contribute to minimizing the environmental impact of the process, ensuring that the application of tantalum coatings remains both economically viable and environmentally sustainable.

## Data Availability

The datasets used and analysed during the current study are available from the corresponding author upon reasonable request.
